# The genome sequence of the cinereous chiton, *Lepidochitona cinerea* (Linnaeus, 1767) (Chitonida: Tonicellidae)

**DOI:** 10.12688/wellcomeopenres.27395.1

**Published:** 2026-08-27

**Authors:** Marine Biological Association Genome Acquisition Lab, Darwin Tree of Life Barcoding Collective, Wellcome Sanger Institute Tree of Life Management, Samples and Laboratory team, Wellcome Sanger Institute Scientific Operations: Sequencing Operations, Wellcome Sanger Institute Tree of Life Core Informatics team, Tree of Life Core Informatics collective, Darwin Tree of Life Consortium

**Keywords:** Lepidochitona cinerea, cinereous chiton, genome sequence, chromosomal, Chitonida

## Abstract

We present a genome assembly from an individual *Lepidochitona cinerea* (cinereous chiton; Mollusca; Polyplacophora; Chitonida; Tonicellidae). The genome sequence has a total length of 705.71 megabases. Most of the assembly (99.68%) is scaffolded into 15 chromosomal pseudomolecules. The mitochondrial genome has also been assembled, with a length of 14.97 kilobases. Gene annotation of this assembly on Ensembl identified 17 286 protein-coding genes. This assembly was generated as part of the Darwin Tree of Life project, which produces genomes for eukaryotic species found in Britain and Ireland.

## Species taxonomy

Eukaryota; Opisthokonta; Metazoa; Eumetazoa; Bilateria; Protostomia; Spiralia; Lophotrochozoa; Mollusca; Polyplacophora; Neoloricata; Chitonida; Acanthochitonina; Tonicellidae; Tonicellinae; *Lepidochitona*; *Lepidochitona cinerea* (Linnaeus, 1767) (NCBI:txid256056).

## Background

*Lepidochitona cinerea* (Linnaeus, 1767), commonly known as the grey chiton, is a marine mollusc in the class Polyplacophora ( MolluscaBase eds., 2026). It has an oval, dorsoventrally flattened body protected by eight overlapping shell valves and surrounded by a narrow girdle. Individuals are usually 12–16 mm long, although specimens of up to 28 mm have been recorded. Despite its common name, the species varies greatly in colour and pattern ( Smith, 2020).

*Lepidochitona cinerea* is the commonest littoral chiton in Britain and occurs from the middle shore through the kelp zone and into subtidal waters. At low tide, it shelters beneath stones, often in rock pools or where stones are partly embedded in sand or gravel. It feeds by scraping microalgae and associated organisms from rock surfaces using a radula with magnetite-hardened teeth. The species is found around Britain and Ireland and more widely from northern Norway to the Mediterranean and Black seas ( Smith, 2020).

We present a chromosome-level genome sequence for *Lepidochitona cinerea*, generated as part of the Darwin Tree of Life Project, which aims to generate high-quality reference genomes for all named eukaryotic species in Britain and Ireland to support research, conservation, and the sustainable use of biodiversity ( Darwin Tree of Life Project Consortium, 2022).

## Methods

### Sample acquisition and DNA barcoding

Three adult *Lepidochitona cinerea* specimens were used: the genome-sequencing specimen (specimen ID MBA-210524-006E, ToLID xpLepCine2; Figure 1), the Hi-C specimen (specimen ID SAN0000305, ToLID xpLepCine1), and the RNA specimen (specimen ID MBA-210525-006A, ToLID xpLepCine7). The genome-sequencing specimen was collected from Barn Pool, Mount Edgcombe, Cornwall, UK (latitude 50.3536, longitude −4.1692) on 2021-05-24 by a team from the Marine Biological Association. The Hi-C specimen was collected from White Bay, Great Cumbrae, North Ayrshire, Scotland, UK (latitude 55.7904, longitude −4.9088) on 2020-08-22 by Richard Durbin (University of Cambridge). The RNA specimen was collected from Churston Cove, Brixham, Devon, UK (latitude 50.4029, longitude −3.524) on 2021-05-25 by a team from the Marine Biological Association.

**Figure 1.**
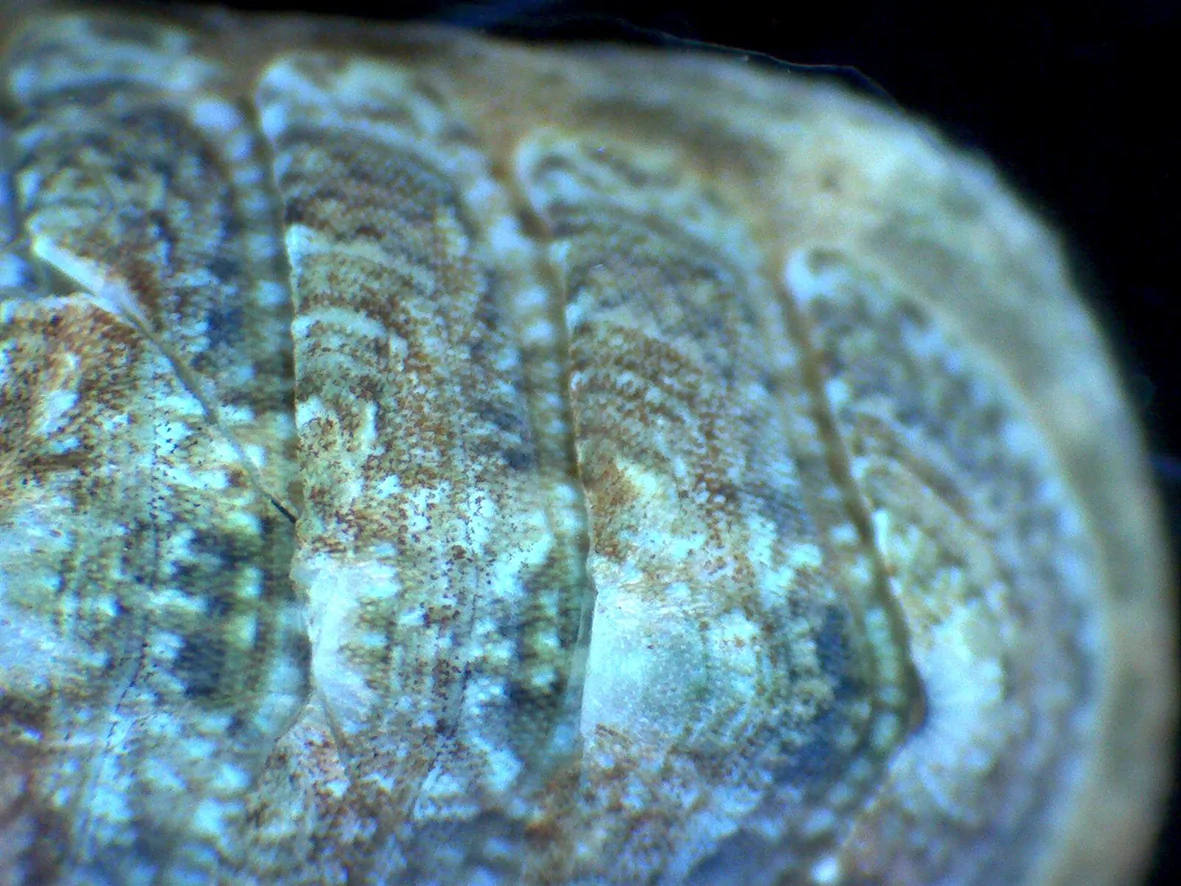
Doral view of the *Lepidochitona cinerea* specimen (MBA-210524-006E; xpLepCine2) used for genome sequencing.

The initial identification was verified by an additional DNA barcoding process according to the framework developed by Twyford *et al.* (2024). A small sample was dissected from the specimen and stored in ethanol, while the remaining parts were shipped on dry ice to the Wellcome Sanger Institute (WSI) (see the protocol). The tissue was lysed, the COI marker region was amplified by PCR, and amplicons were sequenced and compared to the BOLD database. Following whole genome sequence generation, the relevant DNA barcode region was also used alongside the initial barcoding data for sample tracking at the WSI ( Twyford *et al.*, 2024). The standard operating procedures for Darwin Tree of Life barcoding are available on protocols.io.

### Nucleic acid extraction

Detailed protocols for nucleic acid extraction developed at the Wellcome Sanger Institute (WSI) Tree of Life Core Laboratory are available on protocols.io ( Howard *et al.*, 2025). The xpLepCine2 specimen was weighed and triaged to determine the appropriate extraction protocol.

For high molecular weight (HMW) DNA extraction, 26.0 mg of tissue was homogenised by powermashing using a PowerMasher II tissue disruptor. The Automated MagAttract v.1 extraction protocol was used, followed by fragmentation using the Diagenode Megaruptor3 for PacBio HiFi protocol. Sheared DNA was purified by manual SPRI (solid-phase reversible immobilisation). The concentration of the sheared and purified DNA was assessed using a Nanodrop spectrophotometer and Qubit Fluorometer using the Qubit dsDNA High Sensitivity Assay kit. Fragment size distribution was evaluated by running the sample on the FemtoPulse system. For this sample, the final post-shearing DNA had a Qubit concentration of 27.0 ng/μL, with a fragment size of 10.8 kb. The 260/280 spectrophotometric ratio was 1.887, and the 260/230 ratio was 2.027.

RNA was extracted from muscle tissue of the xpLepCine7 specimen in the Tree of Life Laboratory at the WSI using the RNA Extraction: Automated MagMax™ *mir*Vana protocol. The RNA concentration was assessed using a Nanodrop spectrophotometer and a Qubit Fluorometer using the Qubit RNA Broad-Range Assay kit. Analysis of the integrity of the RNA was done using the Agilent RNA 6000 Pico Kit and Eukaryotic Total RNA assay.

### PacBio HiFi library preparation and sequencing

Library preparation and sequencing were performed at the WSI Scientific Operations core. Libraries were prepared using the SMRTbell Prep Kit 3.0 (Pacific Biosciences, California, USA), following the manufacturer’s instructions. The kit includes reagents for end repair/A-tailing, adapter ligation, post-ligation SMRTbell bead clean-up, and nuclease treatment. Size selection and clean-up were performed using diluted AMPure PB beads (Pacific Biosciences). DNA concentration was quantified using a Qubit Fluorometer v4.0 (ThermoFisher Scientific) and the Qubit 1X dsDNA HS assay kit. Final library fragment size was assessed with the Agilent Femto Pulse Automated Pulsed Field CE Instrument (Agilent Technologies) using the gDNA 55 kb BAC analysis kit.

The sample was sequenced using the Sequel IIe system (Pacific Biosciences, California, USA). The concentration of the library loaded onto the Sequel IIe was in the range 40–135 pM. SMRT Link software, a PacBio web-based workflow manager, was used to set up and monitor the run, and to perform primary and secondary analysis of the data upon completion.

### Hi-C

***Sample preparation and crosslinking***

The Hi-C sample was prepared from 20–50 mg of frozen mid-body tissue of the xpLepCine1 sample using the Arima-HiC v2 kit (Arima Genomics). Following the manufacturer’s instructions, tissue was fixed and DNA crosslinked using TC buffer to a final formaldehyde concentration of 2%. The tissue was homogenised using the Diagnocine Power Masher-II. Crosslinked DNA was digested with a restriction enzyme master mix, biotinylated, and ligated. Clean-up was performed with SPRISelect beads before library preparation. DNA concentration was measured with the Qubit Fluorometer (Thermo Fisher Scientific) and Qubit HS Assay Kit. The biotinylation percentage was estimated using the Arima-HiC v2 QC beads.

***Hi-C library preparation and sequencing***

Biotinylated DNA constructs were fragmented using a Covaris E220 sonicator and size selected to 400–600 bp using SPRISelect beads. DNA was enriched with Arima-HiC v2 kit Enrichment beads. End repair, A-tailing, and adapter ligation were carried out with the NEBNext Ultra II DNA Library Prep Kit (New England Biolabs), following a modified protocol where library preparation occurs while DNA remains bound to the Enrichment beads. Library amplification was performed using KAPA HiFi HotStart mix and a custom Unique Dual Index (UDI) barcode set (Integrated DNA Technologies). Depending on sample concentration and biotinylation percentage determined at the crosslinking stage, libraries were amplified with 10–16 PCR cycles. Post-PCR clean-up was performed with SPRISelect beads. Libraries were quantified using the AccuClear Ultra High Sensitivity dsDNA Standards Assay Kit (Biotium) and a FLUOstar Omega plate reader (BMG Labtech).

Prior to sequencing, libraries were normalised to 10 ng/μL. Normalised libraries were quantified again to create equimolar and/or weighted 2.8 nM pools. Pool concentrations were checked using the Agilent 4200 TapeStation (Agilent) with High Sensitivity D500 reagents before sequencing. Sequencing was performed using paired-end 150 bp reads on the Illumina NovaSeq 6000.

### RNA library preparation and sequencing

Libraries were prepared using the NEBNext® Ultra II Directional RNA Library Prep Kit for Illumina (New England Biolabs), following the manufacturer’s instructions. Poly(A) mRNA was isolated from 100 ng of total RNA using oligo (dT) beads, converted to cDNA, and uniquely indexed; 14 PCR cycles were performed. Libraries were prepared with an intended fragment size of 100–300 bp. Libraries were quantified, normalised, and pooled equimolarly to a final concentration of 2.8 nM before dilution to 150 pM for loading. Sequencing was carried out on the Illumina NovaSeq 6000, generating paired-end reads.

### Genome assembly

Prior to assembly of the PacBio HiFi reads, a database of *k*-mer counts ( *k* = 31) was generated from the filtered reads using FastK. GenomeScope2 ( Ranallo-Benavidez *et al.*, 2020) was used to analyse the *k*-mer frequency distributions, providing estimates of genome size, heterozygosity, and repeat content.

The HiFi reads were assembled using Hifiasm ( Cheng *et al.*, 2021) with the --primary option. Haplotypic duplications were identified and removed using purge_dups ( Guan *et al.*, 2020). The Hi-C reads ( Rao *et al.*, 2014) were mapped to the primary contigs using bwa-mem2 ( Vasimuddin *et al.*, 2019), and the contigs were scaffolded in YaHS ( Zhou *et al.*, 2023) with the --break option for handling potential misassemblies. The scaffolded assemblies were evaluated using Gfastats ( Formenti *et al.*, 2022), BUSCO ( Manni *et al.*, 2021) and MerquryFK ( Rhie *et al.*, 2020).

The mitochondrial genome was assembled using MitoHiFi ( Uliano-Silva *et al.*, 2023). The MitoHiFi reference was the mitochondrial genome of *Nuttallina californica* (NC_026849.1).

### Assembly curation

The assembly was decontaminated using the Assembly Screen for Cobionts and Contaminants ( ASCC) pipeline. TreeVal was used to generate the flat files and maps for use in curation. Manual curation was conducted primarily in PretextView and HiGlass ( Kerpedjiev *et al.*, 2018). Scaffolds were visually inspected and corrected as described by Howe *et al*. (2021). Manual corrections included 69 breaks, 61 joins, and removal of 40 haplotypic duplications. This reduced the scaffold count by 37.9%, reduced the scaffold N50 by 16.6%, and reduced the total assembly length by 8.1%. The curation process is described at sanger-tol/curation-resources . PretextSnapshot was used to generate a Hi-C contact map of the final assembly.

### Assembly quality assessment

The MerquryFK tool ( Rhie *et al.*, 2020) was run in a Singularity container ( Kurtzer *et al.*, 2017) to evaluate *k*-mer completeness and assembly quality for the primary and alternate haplotypes using the *k*-mer database ( *k* = 31) computed prior to genome assembly. The analysis outputs included assembly QV scores and completeness statistics.

The genome was analysed using the BlobToolKit pipeline, a Nextflow implementation of the earlier Snakemake version ( Challis *et al.*, 2020). PacBio reads were aligned to the assembly using minimap2 ( Li, 2018) and processed with SAMtools ( Danecek *et al.*, 2021) to generate coverage tracks. The pipeline runs BUSCO ( Manni *et al.*, 2021) using lineages identified from the NCBI Taxonomy ( Schoch *et al.*, 2020). For the three basal BUSCO lineages, eukaryota, bacteria and archaea, BUSCO genes are aligned to the UniProt Reference Proteomes database ( Bateman *et al.*, 2023) using DIAMOND BLASTp ( Buchfink *et al.*, 2021). The genome assembly is divided into chunks based on the density of BUSCO genes from the closest taxonomic lineage, and each chunk is aligned to the UniProt Reference Proteomes database using DIAMOND BLASTx. Sequences without DIAMOND BLASTx hits are extracted using seqtk and aligned to the NT database using BLASTn ( Altschul *et al.*, 1990). The outputs are consolidated into a BlobDir for visualisation in BlobToolKit. The BlobToolKit pipeline was developed using nf-core tooling ( Ewels *et al.*, 2020) and MultiQC ( Ewels *et al.*, 2016), with containerisation through Docker ( Merkel, 2014) and Singularity ( Kurtzer *et al.*, 2017).

## Genome sequence report

### Sequence data

PacBio sequencing of the *Lepidochitona cinerea* specimen generated 48.10 Gb (gigabases) from 5.41 million reads, which were used to assemble the genome. GenomeScope2.0 analysis using a *p* = 2 model gave a *k*-mer-based genome size estimate of 695.06 Mb, with a heterozygosity of 3.19% and repeat content of 47.80% ( Figure 2). These estimates guided expectations for the assembly. Based on the estimated genome size, the sequencing data provided approximately 65× coverage. Hi-C sequencing produced 355.14 Gb from 1 175.95 million reads, which were used to scaffold the assembly. RNA sequencing data were also generated and are available in public sequence repositories. Table 1 summarises the specimen and sequencing details.

**Figure 2.**
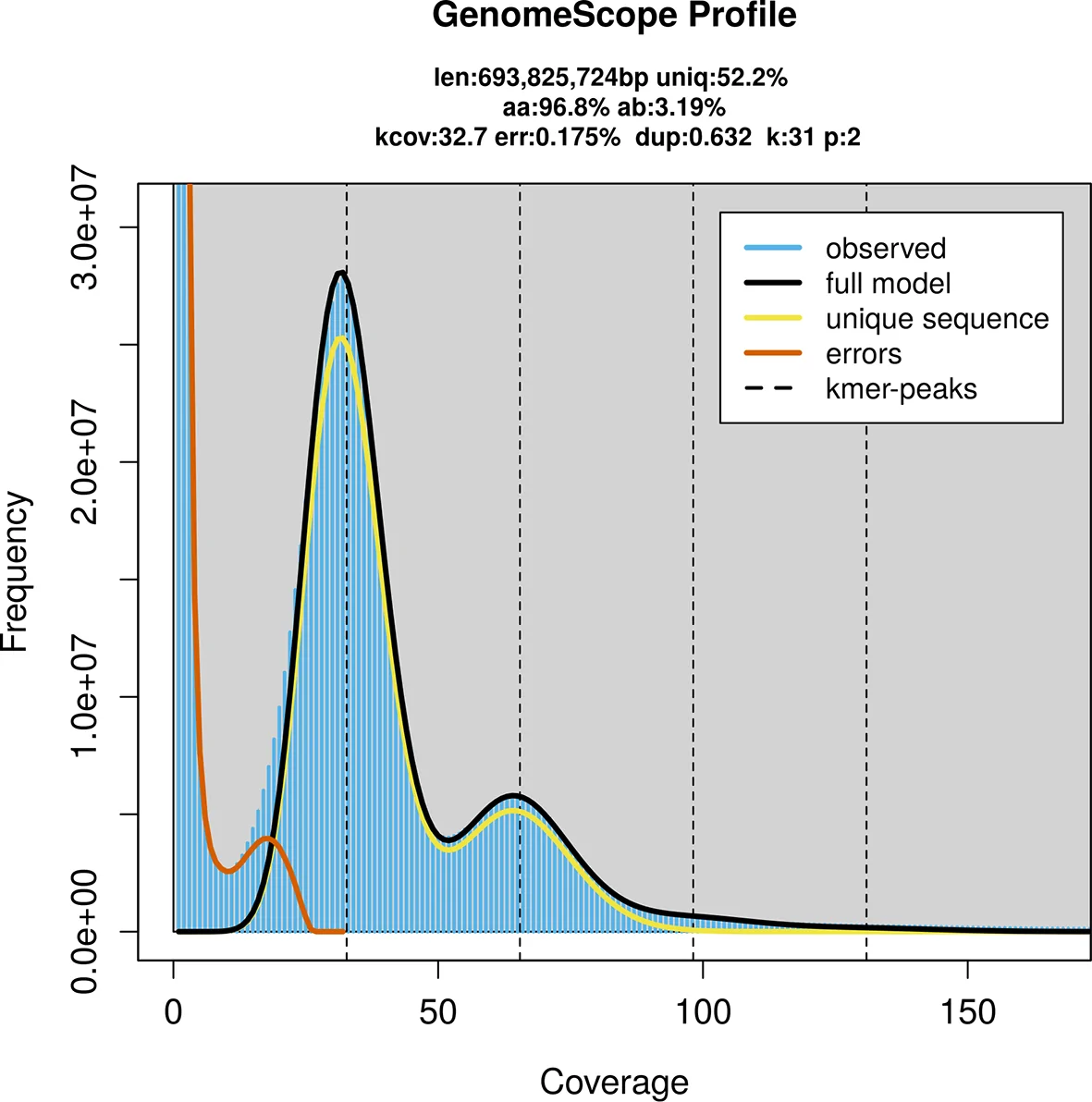
Frequency distribution of *k*-mers generated using GenomeScope2. The plot shows observed and modelled *k*-mer spectra, providing estimates of genome size, heterozygosity, and repeat content based on unassembled sequencing reads.

**Table 1.** Specimen and sequencing data for *Lepidochitona cinerea* (BioProject PRJEB72111).

Platform	PacBio HiFi	Hi-C	RNA-seq
**ToLID**	xpLepCine2	xpLepCine1	xpLepCine7
**Specimen ID**	MBA-210524-006E	SAN0000305	MBA-210525-006A
**BioSample (source individual)**	SAMEA12219462	SAMEA7522718	SAMEA12219467
**BioSample (tissue)**	SAMEA12219717	SAMEA7522765	SAMEA12219723
**Tissue**	other somatic animal tissue	mid body	muscle
**Instrument**	Sequel IIe	Illumina NovaSeq 6000	Illumina NovaSeq 6000
**Run accessions**	ERR12535451; ERR12535452	ERR12542994; ERR12542988	ERR12542993
**Read count total**	5.41 million reads	1 175.95 million read pairs	30.71 million read pairs
**Base count total**	48.10 Gb	355.14 Gb	9.27 Gb

### Assembly statistics

The primary haplotype was assembled, and contigs corresponding to an alternate haplotype were also deposited in INSDC databases. The final assembly has a total length of 705.71 Mb in 86 scaffolds, with 134 gaps, and a scaffold N50 of 46.32 Mb ( Table 2).

**Table 2.** Genome assembly data for *Lepidochitona cinerea.*

Genome assembly	Primary assembly
**Assembly name**	xpLepCine2.1
**Assembly accession**	GCA_963971465.1
**Alternate haplotype accession**	GCA_963971505.1
**Assembly level**	chromosome
**Span (Mb)**	705.71
**Number of chromosomes**	15
**Number of contigs**	220
**Contig N50**	8.73 Mb
**Number of scaffolds**	86
**Scaffold N50**	46.32 Mb
**Organelles**	Mitochondrial genome: 14.97 kb

Most of the assembly sequence (99.68%) was assigned to 15 chromosomal-level scaffolds. These chromosome-level scaffolds, confirmed by Hi-C data, are named according to size ( Figure 3; Table 3). In genome curation we observed that chromosome 4 has substantial heterozygous structural variation with breakpoints between haplotypes at approximately 14.1 Mb, 29 Mb and 35.9 Mb. We also noted that chromosome 9 has a heterozygous inversion between 8.9 Mb and 17 Mb.

**Figure 3.**
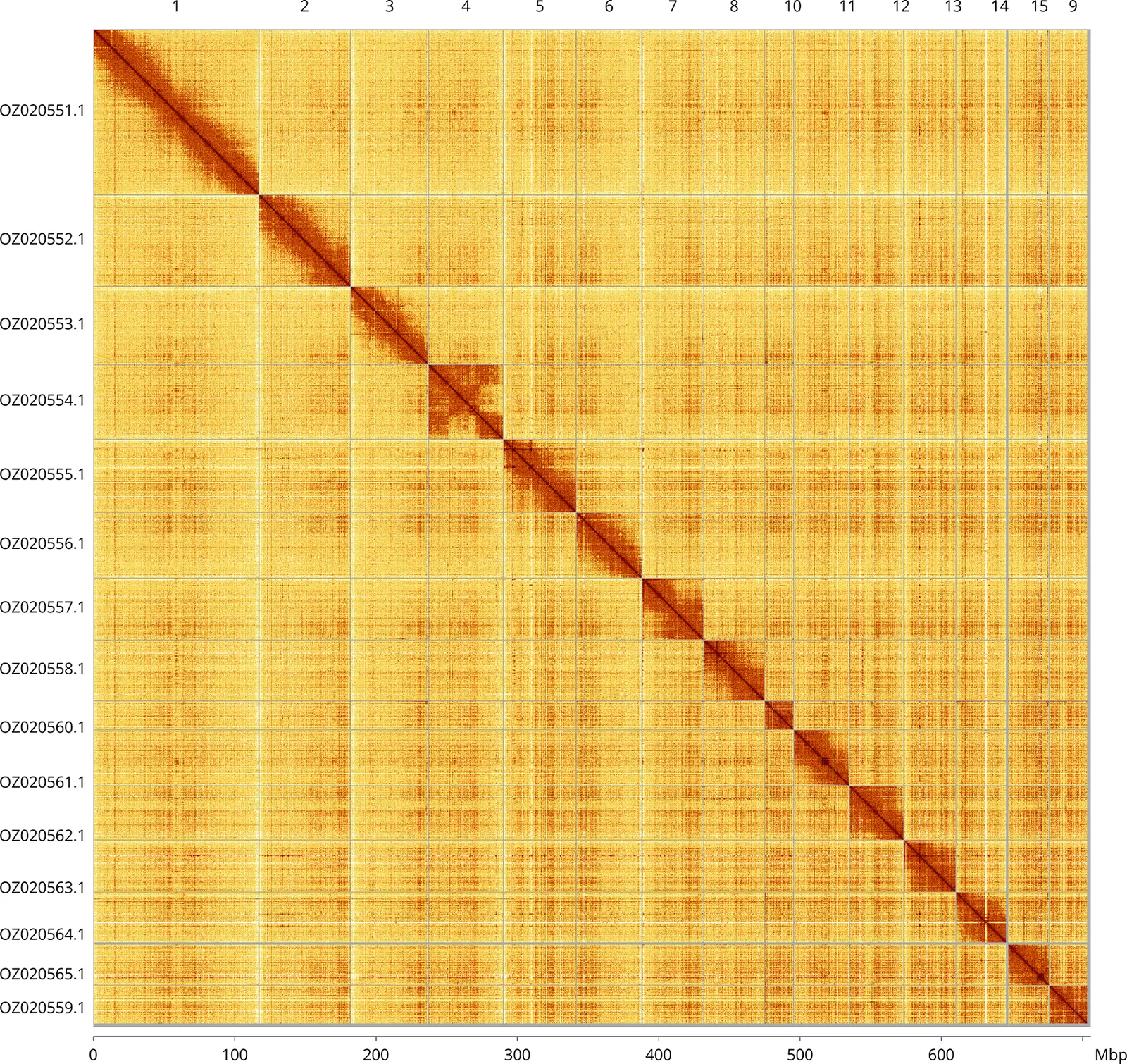
Hi-C contact map of the *Lepidochitona cinerea* genome assembly. Assembled chromosomes are shown in order of size and labelled along the axes, with a megabase scale shown below. The plot was generated using PretextSnapshot.

**Table 3.** Chromosomal pseudomolecules in the primary genome assembly of *Lepidochitona cinerea* xpLepCine2.

INSDC accession	Molecule	Length (Mb)	GC%
OZ020551.1	1	117.21	37.50
OZ020552.1	2	64.88	37.50
OZ020553.1	3	55.06	37.50
OZ020554.1	4	52.88	37.50
OZ020555.1	5	51.79	38
OZ020556.1	6	46.32	37.50
OZ020557.1	7	43.81	38
OZ020558.1	8	43.16	37.50
OZ020559.1	9	20.39	38.50
OZ020560.1	10	39.51	37.50
OZ020561.1	11	38.40	38
OZ020562.1	12	36.89	38
OZ020563.1	13	36.86	38
OZ020564.1	14	29.06	38
OZ020565.1	15	27.25	38

The mitochondrial genome was also assembled (length 14.97 kb, OZ020566.1). This sequence is included as a contig in the multifasta file of the genome submission and as a standalone record.

### Assembly quality metrics

The combined primary and alternate assemblies achieve an estimated QV of 64.0. The *k*-mer completeness is 61.04% for the primary assembly, 58.66% for the alternate haplotype, and 96.10% for the combined assemblies ( Figure 4).

**Figure 4.**
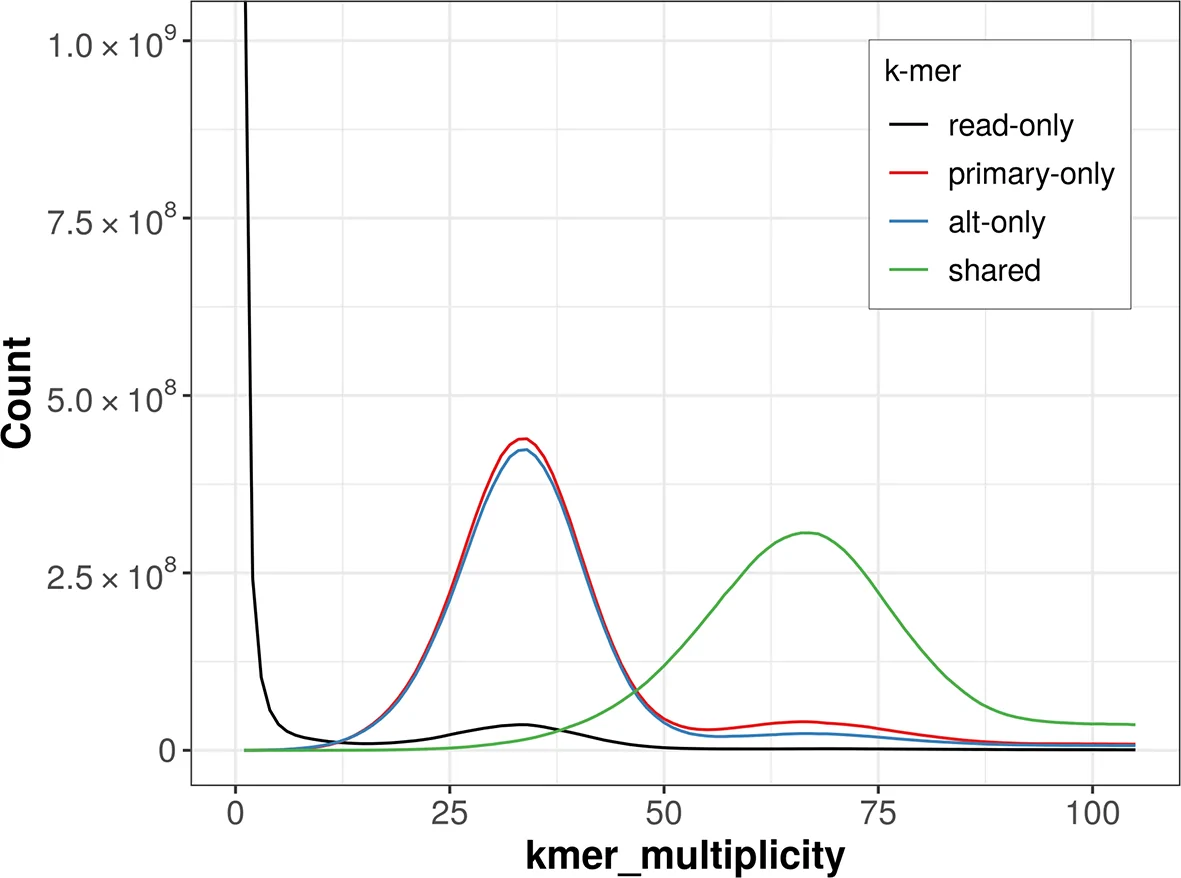
Evaluation of *k*-mer completeness using MerquryFK. This plot illustrates the recovery of *k*-mers from the original read data in the final assemblies. The horizontal axis represents *k*-mer multiplicity, and the vertical axis shows the number of *k*-mers. The black curve represents *k*-mers that appear in the reads but are not assembled. The green curve corresponds to *k*-mers shared by both haplotypes, and the red and blue curves show *k*-mers found only in one of the haplotypes.

BUSCO v.5.5.0 analysis using the mollusca_odb10 reference set ( *n* = 5 295) identified 78.1% of the expected gene set (single = 77.2%, duplicated = 0.9%). The snail plot in Figure 5 summarises the scaffold length distribution and other assembly statistics for the primary assembly. The blob plot in Figure 6 shows the distribution of scaffolds by GC proportion and coverage.

**Figure 5.**
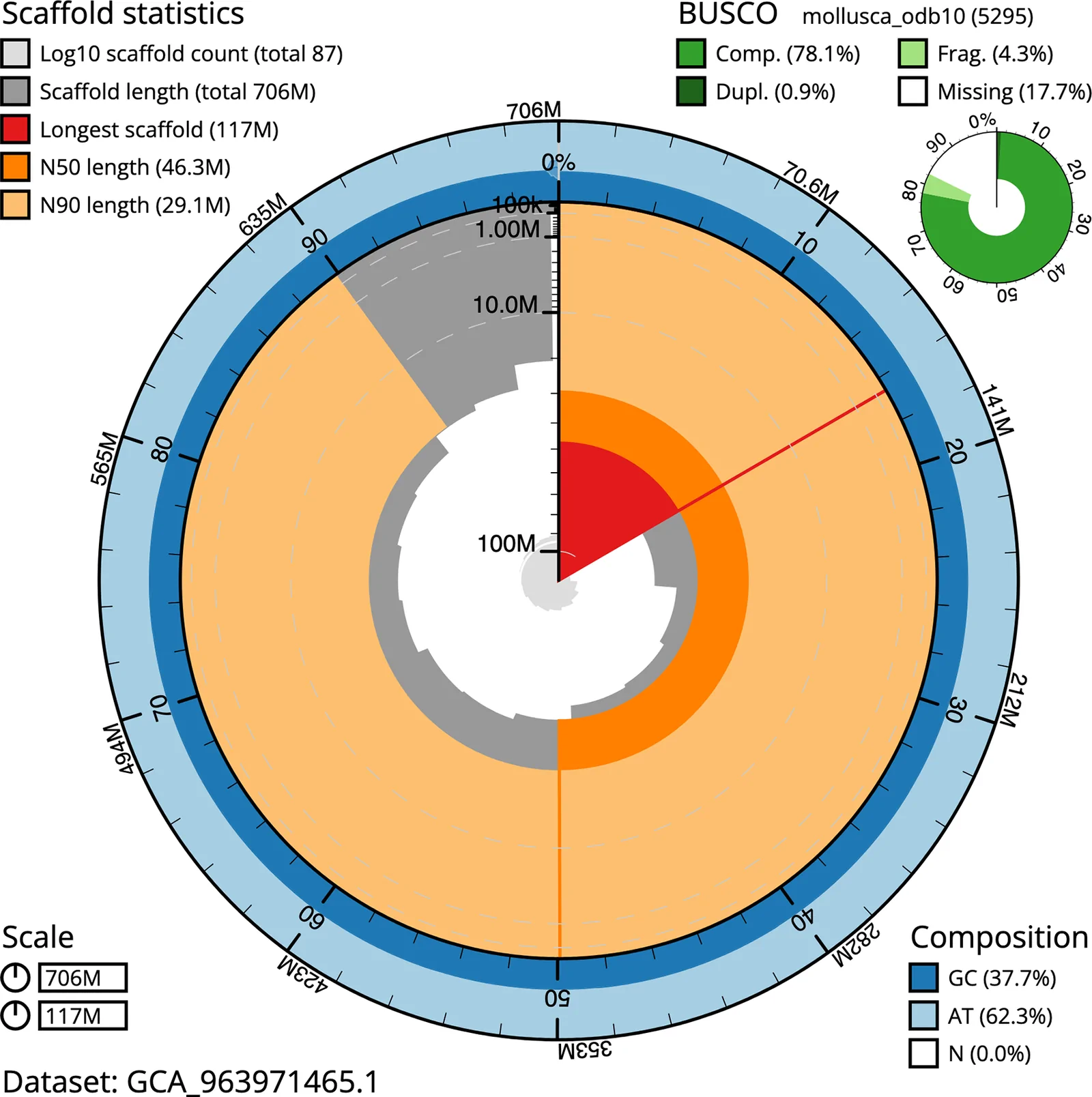
Assembly metrics for xpLepCine2.1. The BlobToolKit snail plot provides an overview of assembly metrics and BUSCO gene completeness. The circumference represents the length of the whole genome sequence, and the main plot is divided into 1 000 bins around the circumference. The outermost blue tracks display the distribution of GC, AT, and N percentages across the bins. Scaffolds are arranged clockwise from longest to shortest and are depicted in dark grey. The longest scaffold is indicated by the red arc, and the deeper orange and pale orange arcs represent the N50 and N90 lengths. A light grey spiral at the centre shows the cumulative scaffold count on a logarithmic scale. A summary of complete, fragmented, duplicated, and missing BUSCO genes in the mollusca_odb10 set is presented at the top right. An interactive version of this figure can be accessed on the BlobToolKit viewer.

**Figure 6.**
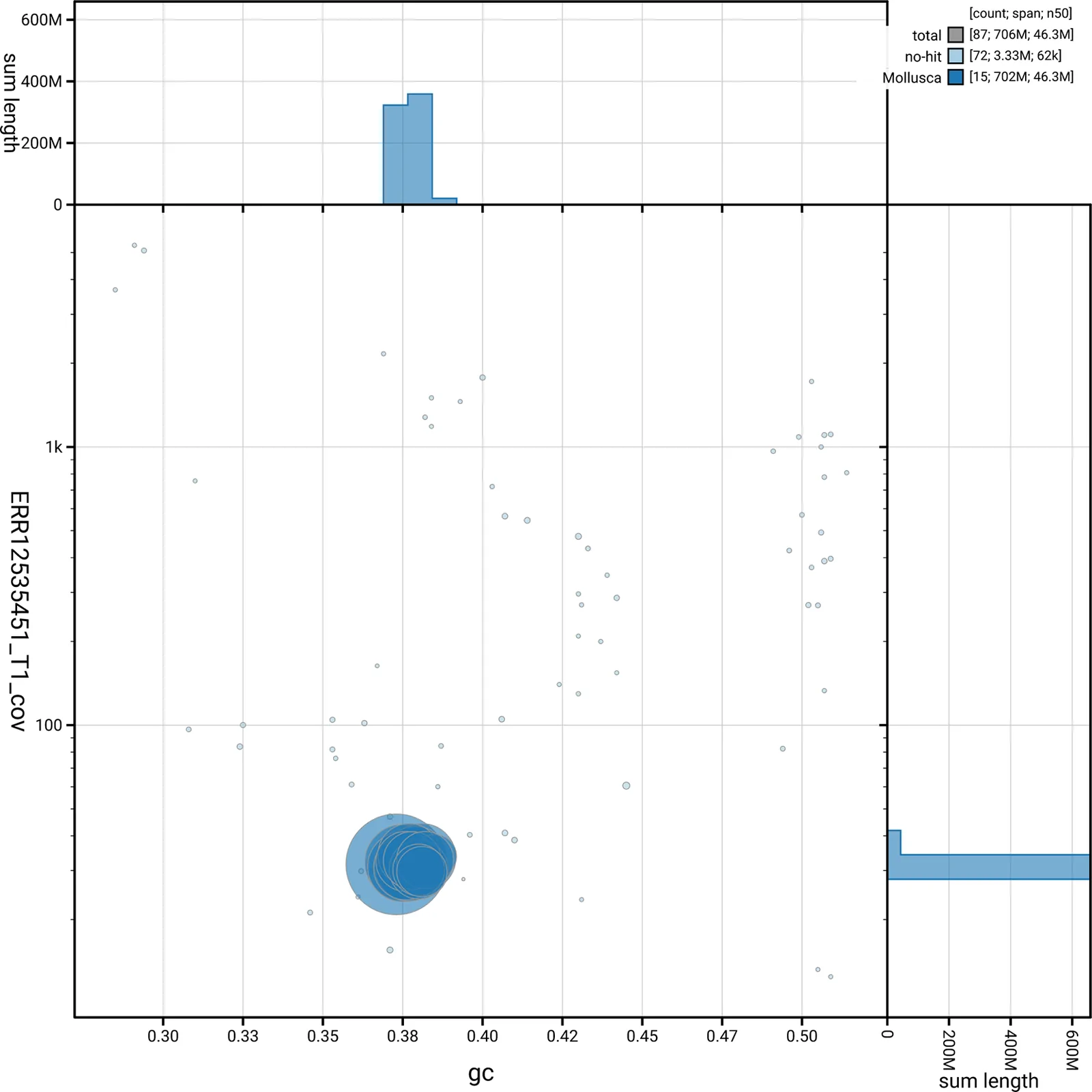
BlobToolKit blob plot for xpLepCine2.1. The plot shows base coverage (vertical axis) and GC content (horizontal axis). The circles represent scaffolds, with the size proportional to scaffold length and the colour representing phylum membership. The histograms along the axes display the total length of sequences distributed across different levels of coverage and GC content. An interactive version of this figure is available on the BlobToolKit viewer.

Table 4 lists the assembly metric benchmarks adapted from Rhie *et al.* (2021) and the Earth BioGenome Project (EBP) Report on Assembly Standards. The EBP metric, calculated for primary assembly, is **6.C.Q65**, meeting the recommended 6.C.Q40 reference standard.

**Table 4.** Earth Biogenome Project summary metrics for the *Lepidochitona cinerea* assembly.

Measure	Value	Benchmark
EBP summary (primary)	6.C.Q65	6.C.Q40
Contig N50 length	8.73 Mb	≥ 1 Mb
Scaffold N50 length	46.32 Mb	= chromosome N50
Consensus quality (QV)	Primary: 65.3; alternate: 63.4; combined: 64.0	≥ 40
*k*-mer completeness	Primary: 61.04%; alternate: 58.66%; combined: 96.10%	> 90%
BUSCO	C:78.1% [S:77.2%, D:0.9%], F:4.3%, M:17.7%, n:5 295	S > 90%; D < 5%
Percentage of assembly assigned to chromosomes	99.68%	≥ 90%

**Notes:** The EBP summary uses log10(Contig N50); chromosome-level (C) or log10(Scaffold N50); Q (Merqury QV). BUSCO: C = complete; S = single-copy; D = duplicated; F = fragmented; M = missing; n = orthologues.

**Table 5.** Software versions and sources used for *Lepidochitona cinerea.*

Software	Version	Source
BLAST	2.15.0+	https://ftp.ncbi.nlm.nih.gov/blast/executables/blast+/
BlobToolKit	4.3.9	https://github.com/blobtoolkit/blobtoolkit
BUSCO	5.5.0	https://gitlab.com/ezlab/busco
bwa-mem2	2.2.1	https://github.com/bwa-mem2/bwa-mem2
DIAMOND	2.1.8	https://github.com/bbuchfink/diamond
fasta_windows	0.2.4	https://github.com/tolkit/fasta_windows
FastK	1.1	https://github.com/thegenemyers/FASTK
GenomeScope2.0	2.0.1	https://github.com/tbenavi1/genomescope2.0
Gfastats	1.3.6	https://github.com/vgl-hub/gfastats
Hifiasm	0.16.1	https://github.com/chhylp123/hifiasm
HiGlass	1.13.4	https://github.com/higlass/higlass
MerquryFK	1.1.0-c1	https://github.com/thegenemyers/MERQURY.FK
Minimap2	2.24-r1122; 2.28-r1209	https://github.com/lh3/minimap2
MitoHiFi	3.2	https://github.com/marcelauliano/MitoHiFi
MultiQC	1.14; 1.17 and 1.18	https://github.com/MultiQC/MultiQC
Nextflow	23.04.1; 24.10.4	https://github.com/nextflow-io/nextflow
PretextSnapshot	0.0.6	https://github.com/sanger-tol/PretextSnapshot
PretextView	1.0.3	https://github.com/sanger-tol/PretextView
purge_dups	1.2.3	https://github.com/dfguan/purge_dups
samtools	1.16.1; 1.17; 1.18; 1.20; 1.21	https://github.com/samtools/samtools
sanger-tol/ascc	0.1.0	https://github.com/sanger-tol/ascc
sanger-tol/blobtoolkit	0.4.0	https://github.com/sanger-tol/blobtoolkit
sanger-tol/curationpretext	1.4.2	https://github.com/sanger-tol/curationpretext
Seqtk	1.4-r122	https://github.com/lh3/seqtk
Singularity	3.9.0	https://github.com/sylabs/singularity
TreeVal	1.0.2	https://github.com/sanger-tol/treeval
YaHS	1.1a.2	https://github.com/c-zhou/yahs

### Genome annotation report

The *Lepidochitona cinerea* genome assembly (GCA_963971465.1) was annotated by Ensembl at the European Bioinformatics Institute (EBI). This annotation includes 64 519 transcribed mRNAs from 17 286 protein-coding and 36 710 non-coding genes. The average transcript length is 7 767.21 bp, with an average of 1.19 coding transcripts per gene and 4.59 exons per transcript. Further details of this annotation are available from the Ensembl annotation page.

## Author information

Contributors are listed at the following links:

• Members of the Marine Biological Association Genome Acquisition Lab
• Members of the Darwin Tree of Life Barcoding collective
• Members of the Wellcome Sanger Institute Tree of Life Management, Samples and Laboratory team
• Members of Wellcome Sanger Institute Scientific Operations – Sequencing Operations
• Members of the Wellcome Sanger Institute Tree of Life Core Informatics team
• Members of the Tree of Life Core Informatics collective
• Members of the Darwin Tree of Life Consortium

## Wellcome sanger institute – legal and governance

The materials that have contributed to this genome note have been supplied by a Darwin Tree of Life Partner. The submission of materials by a Darwin Tree of Life Partner is subject to the **‘Darwin Tree of Life Project Sampling Code of Practice’**, which can be found in full on the Darwin Tree of Life website. By agreeing with and signing up to the Sampling Code of Practice, the Darwin Tree of Life Partner agrees they will meet the legal and ethical requirements and standards set out within this document in respect of all samples acquired for, and supplied to, the Darwin Tree of Life Project. Further, the Wellcome Sanger Institute employs a process whereby due diligence is carried out proportionate to the nature of the materials themselves, and the circumstances under which they have been/are to be collected and provided for use. The purpose of this is to address and mitigate any potential legal and/or ethical implications of receipt and use of the materials as part of the research project, and to ensure that in doing so we align with best practice wherever possible. The overarching areas of consideration are:

• Ethical review of provenance and sourcing of the material
• Legality of collection, transfer and use (national and international)

Each transfer of samples is further undertaken according to a Research Collaboration Agreement or Material Transfer Agreement entered into by the Darwin Tree of Life Partner, Genome Research Limited (operating as the Wellcome Sanger Institute), and in some circumstances, other Darwin Tree of Life collaborators.

## Data Availability

European Nucleotide Archive: Lepidochitona cinerea (grey chiton). Accession number PRJEB72111; https://identifiers.org/ena.embl/PRJEB72111. The genome sequence is released openly for reuse. The *Lepidochitona cinerea* genome sequencing initiative is part of the Darwin Tree of Life Project (PRJEB40665) and the Sanger Institute Tree of Life Programme (PRJEB43745). All raw sequence data and the assembly have been deposited in INSDC databases. Raw data and assembly accession identifiers are reported in Tables 1 and 2. Production code used in genome assembly at the WSI Tree of Life is available at https://github.com/sanger-tol . Table 5 lists software versions used in this study.
