# Supplementary material for: The genome sequence of the cinereous chiton, Lepidochitona cinerea (Linnaeus, 1767) (Chitonida: Tonicellidae)
Source: Wellcome Open Res. 2026 Aug 27;11:550. [Version 1] doi: 10.12688/wellcomeopenres.27395.1 (PMC13736060; doi:10.12688/wellcomeopenres.27395.1)
Supplement: Peer review history

# Reviewer report: Reviewer response for version 1

**DOI:** 10.21956/wellcomeopenres.30152.r175669

**Published:** 2026-11-04

**Authors:** Adrián Jaimes-Becerra

**Affiliations:** 1 Tohoku University, Sendai, Japan

This Data Note presents a valuable chromosome-scale reference genome for the marine chiton Lepidochitona cinerea. The primary assembly spans 705.71 Mb, with 99.68% of the sequence assigned to 15 chromosomal pseudomolecules. I only have two comments.

 -The HiFi and Hi-C data came from different individuals. Could they influence the interpretation of structural variation?

 -Could the authors share the barcode sequences or their accession numbers so readers can verify the identifications?

Are sufficient details of methods and materials provided to allow replication by others?

Yes

Is the rationale for creating the dataset(s) clearly described?

Yes

Are the datasets clearly presented in a useable and accessible format?

Yes

Are the protocols appropriate and is the work technically sound?

Yes

Reviewer Expertise:

Evolutionary biology, Cnidaria genomics, venom evolution

I confirm that I have read this submission and believe that I have an appropriate level of expertise to confirm that it is of an acceptable scientific standard.

# Reviewer report: Reviewer response for version 1

**DOI:** 10.21956/wellcomeopenres.30152.r166191

**Published:** 2026-09-11

**Authors:** Erich M Schwarz

**Affiliations:** 1 Cornell University, Ithaca, New York, USA

The paper is generally satisfactory.  The one notable oddity was the assembly of chromosome 4 (OZ020554.1), which in the Hi-C heat map looks almost as if its 3' and 5' arms could be assembled into a credible chromosome if the central region were left out as a separate small chromosome.  Since the authors used a careful manual revision of the original Hi-C scaffolding (via YaHS) and observed what they describe as structural polymorphisms, I assume that this odd heatmap for chromosome 4 represents their best judgement about how this should be scaffolded.

Are sufficient details of methods and materials provided to allow replication by others?

Yes

Is the rationale for creating the dataset(s) clearly described?

Yes

Are the datasets clearly presented in a useable and accessible format?

Yes

Are the protocols appropriate and is the work technically sound?

Yes

Reviewer Expertise:

Nematode genomics

I confirm that I have read this submission and believe that I have an appropriate level of expertise to confirm that it is of an acceptable scientific standard.
